# Speed and direction changes induce the perception of animacy in 7-month-old infants

**DOI:** 10.3389/fpsyg.2014.01141

**Published:** 2014-10-10

**Authors:** Birgit Träuble, Sabina Pauen, Diane Poulin-Dubois

**Affiliations:** ^1^Department of Psychology, University of CologneCologne, Germany; ^2^Department of Psychology, Heidelberg UniversityHeidelberg, Germany; ^3^Department of Psychology, Concordia UniversityMontréal, QC, Canada

**Keywords:** infancy, animacy perception, animate–inanimate distinction, category-specific motion

## Abstract

A large body of research has documented infants’ ability to classify animate and inanimate objects based on static or dynamic information. It has been shown that infants less than 1 year of age transfer animacy-specific expectations from dynamic point-light displays to static images. The present study examined whether basic motion cues that typically trigger judgments of perceptual animacy in older children and adults lead 7-month-olds to infer an ambiguous object’s identity from dynamic information. Infants were tested with a novel paradigm that required inferring the animacy status of an ambiguous moving shape. An ambiguous shape emerged from behind a screen and its identity could only be inferred from its motion. Its motion pattern varied distinctively between scenes: it either changed speed and direction in an animate way, or it moved along a straight path at a constant speed (i.e., in an inanimate way). At test, the identity of the shape was revealed and it was either consistent or inconsistent with its motion pattern. Infants looked longer on trials with the inconsistent outcome. We conclude that 7-month-olds’ representations of animates and inanimates include category-specific associations between static and dynamic attributes. Moreover, these associations seem to hold for simple dynamic cues that are considered minimal conditions for animacy perception.

## INTRODUCTION

To recognize a given entity as being either animate or inanimate is thought to be a fundamental process characterizing human perception and cognition (e.g., Opfer and Gelman, 2010). Furthermore, the perception of animacy has been hypothesized to relate to the broader domain of social cognition (Pavlova, 2011, 2013; Kaiser and Shiffrar, 2013). Many developmental studies have shown that infants differentiate between these global categories within the first year of life (e.g., Mandler and McDonough, 1993, 1998; Behl-Chadha, 1996; Quinn and Johnson, 2000; Pauen, 2002). Being interested in what kind of information infants may use for developing such broad categories, a large body of research explores the role of *static information*. Among those static attributes considered relevant for identifying animates in terms of their appearance are facial features (e.g., Smith and Heise, 1992; Johnson et al., 1998; Johnson, 2000), leg-like appendages (Mandler and McDonough, 1993, 1998; Rakison, 2005), body configuration (e.g., Eimas and Quinn, 1994; Eimas et al., 1994), and surface information (Johnson et al., 1998; Woodward et al., 2001).

Other studies have explored infants’ knowledge of properties of animates and inanimates based on *dynamic information*. Presenting point-light displays which show only the movement pattern but not the appearance of objects, Bertenthal et al. (1984) demonstrated that 4-month-olds distinguish biological from mechanical motion (see also Bertenthal et al., 1985). Arterberry and Bornstein (2001) found that infants as young as 3 months were able to categorize animals and vehicles based on point-light displays.

Some other work has focused on dynamic attributes which are defined in causal or functional terms. Among those behavioral properties which seem crucial for identifying animate beings are goal-directedness (e.g., Gergely et al., 1995; Rochat et al., 1997; Shimizu and Johnson, 2004; Luo and Baillargeon, 2005; Schlottmann and Ray, 2010) and self-propelledness. For example, even 7-month-olds expect only humans to be self-propelled (e.g., Poulin-Dubois et al., 1996; Saxe et al., 2005; Markson and Spelke, 2006; Pauen and Träuble, 2009). In sum, developmental research clearly suggests that infants less than one year of age are well able to distinguish animals and inanimate objects according to their motion behavior, as well as their appearance in categorical terms.

Being interested in how infants become able to relate specific motion pattern to category-specific behavioral features, numerous studies have focused on infants’ attribution of goals to biologically moving entities (e.g., Shimizu and Johnson, 2004; Luo and Baillargeon, 2005; Schlottmann and Ray, 2010). Corresponding data suggests that young infants attribute goals even to schematic shapes if presented in animate motion (typically operationalized as non-rigid, caterpillar motion, or self-propulsion).

To date only a few studies have addressed the question of how infants become able to relate specific motion pattern to category-specific appearance and to infer one from the other. Pauen and Träuble (2009) tested whether 7-month-old infants consider category-boundaries (animate, inanimate entities) when ascribing self-propelledness within an ambiguous motion event. Infants were shown an unfamiliar animal and a ball, physically connected to each other and performing a contingent animate-like motion on a small stage (i.e., self-propelled, following a non-linear path; see Mandler, 2004). When both entities were later presented motionless in separate locations, infants expected the animal and not the ball to start moving again. Because the spatiotemporal information was ambiguous (i.e., both objects showed the same type of motion), infants had to consider dispositional status information in order to ascribe the origin of motion to one of the objects. Consistent with this interpretation, a follow-up study by the same authors revealed that infants no longer showed looking preferences for the animal when static features characteristic for the appearance of animate beings were either removed (i.e., facial features) or replaced by inanimate features (i.e., furry body replaced by a plastic spiral). This pattern of results demonstrates that 7-months-old infants can form category-specific relations between static and dynamic attributes and that infants attend to static information (facial and body features) about the dispositional status of the entities in order to identify the “motion-originator” in a spatiotemporally ambiguous motion event.

While both types of information were provided simultaneously in the Pauen and Träuble (2009) paradigm, Arterberry and Bornstein (2002) were interested in infants’ ability to *transfer* their expectations from dynamic to static displays of animates and inanimates and vice versa. Differing from Pauen and Träuble (2009), the dynamic displays were realized as point light displays, thereby conveying reduced shape information. The authors provided 6- and 9-month-olds with either pictures or with dynamic point-light-displays showing different exemplars of the same global category (either animals or vehicles) for familiarization. At test, one new exemplar of the familiar category was combined with one exemplar of the contrasting category. When the same kind of presentation format was used during familiarization and at test, even 6-month-olds showed a positive categorization response, hence suggesting that static or dynamic cues can both provide the basis for categorizing animals and vehicles at an early age. When infants were familiarized with point-light-displays and saw pictures at test, 6-month-olds failed, but 9-month-olds succeeded in discriminating both global categories.

It should be noticed that point-light displays are created by highlighting the joints of the entities to be studied, they provide implicit shape information (Cutting, 1978; Giese and Poggio, 2003; Schlottmann and Ray, 2010). Hence, Pauen and Träuble (2009), as well as Arterberry and Bornstein (2002) provided infants with characteristic shape information about the entities shown in motion. In the present study we asked whether 7-month-olds are able to use markedly reduced dynamic information about motion path to infer the identity of a morphologically ambiguous entity.

In the literature on perceptual animacy, numerous studies have manipulated the motion of simple objects, such as geometric shapes (see Gelman and Opfer, 2002; Rutherford et al., 2006; for reviews, see Scholl and Tremoulet, 2000; Gyulai, 2004). The rationale for this work is that when the identity of an object is ambiguous, animacy can be detected by using motion cues. Blythe et al. (1999) have argued that a small set of motion cues can be sufficient not only to determine whether or not a moving object is animate, but also to determine what intention motivated the object’s movement. Tremoulet and Feldman (2000) examined human perception of animacy, based on the motion of simple geometric shapes. In their study, adult participants responded to displays of single objects moving across an otherwise empty background. Their experiments revealed that even very simple motions can serve as effective cues to our perception of animacy; more specifically, changes in speed and direction seem to be crucial for perceiving a given entity as animate (especially when adding orientation changes in the sense that the object’s principal axis is always aligned with the direction of motion). Schultz and Bülthoff (2013) have shown that a single-dot stimulus can evoke the impression of a natural animate entity. Using a parametric design, the authors induced a gradual variation in the percept of animacy by systemically manipulating the impression of self-propelledness. Changes in this movement parameter led to gradual animacy judgments in adult participants without resorting to additional information about form or interaction between objects.

Previous studies suggest an early sensitivity to some dynamic cues to animacy (e.g., self-propulsion). However, infants’ sensitivity to the specific cues that trigger animacy perception in adults has not yet been studied systematically. We do not yet know whether information about the speed constancy and direction of a given object’s motion is sufficient to make infants, like adults, to form expectations regarding the animacy status of a given entity. The present study addresses this issue. For this purpose, we developed a paradigm that requires infants to infer the identity of a moving “shadow,” a gray colored silhouette whose ambiguous shape does not reveal its identity. Speed constancy, motion direction, and orientation of the shadow’s motion were distinctively varied to disambiguate the animacy status of the target entity before it was finally revealed during the test phase. We tested 7-month-olds, based on the findings of Pauen and Träuble (2009) who demonstrated that infants at that age associate self-propelled motion with animals but not with inanimate objects. If 7-month-old infants can infer the identity of the shadow based on motion cues alone, we expected them to look longer at test trials revealing inconsistency between appearance information and previous motion information regarding the target entity.

### ETHICS STATEMENT

All experiments reported in this manuscript were conducted at a German University where institutional review boards or committees are not mandatory, but where researchers need to follow the rules and regulations of the code of conduct for good scientific practice (http://www.uni-heidelberg.de/universitaet/profil/wissenschaftliche_praxis/). The study complies with the APA ethical standards.

The experiments consisted in non-invasive and unconstrained behavioral observations of infants. Prior to the experimental session, parents were given full information about the procedure and the duration of the experiment. Only infants, for whom parental informed consent was obtained, participated in the study. The data were analyzed anonymously.

## EXPERIMENT 1

### METHOD

The basic principle of the shadow paradigm is to present infants with two entities casting identical shadows when hidden behind a semi-opaque screen (phase 1) before showing only one shadow moving either in an animate or an inanimate way (phase 2). At test (phase 3) the semi-opaque screen is removed to reveal one object whose motion was either consistent or inconsistent with its animacy status. We predicted that 7-month-olds would look longer at inconsistent than at consistent trials. To test this assumption, we used a within-subject design.

### PARTICIPANTS

A total of 35 7-month-old infants (*M* = 7 months, 13 days; range = 7 months, 1 day to 7 months, 29 days), 18 girls and 17 boys participated. 10 additional infants had to be excluded due to fussiness (*n* = 2), not looking at least 3 s at the critical motion event during phase 2 (*n* = 2), or failing to look during all four test trials during phase 3 (*n* = 6). All infants came from a White, middle-class socioeconomic background.

### MATERIALS

The stimuli were two animate beings (dog, cow), and two inanimate objects (truck, motorcycle), each presented as realistic 2D photographs. We chose mammals and vehicles because exemplars of both types have a comparably complex appearance as well as parts associated with movement. Perhaps most importantly, identical blurred shadows in terms of overall shape and size could be created when both objects were covered by a semi-lucent screen. The dog was always paired with the truck, and the cow was paired with the motorcycle. The two entities were presented in computer-animated scenes (created with Microsoft® Power Point®).

### DESIGN

**FIGURE 1:**
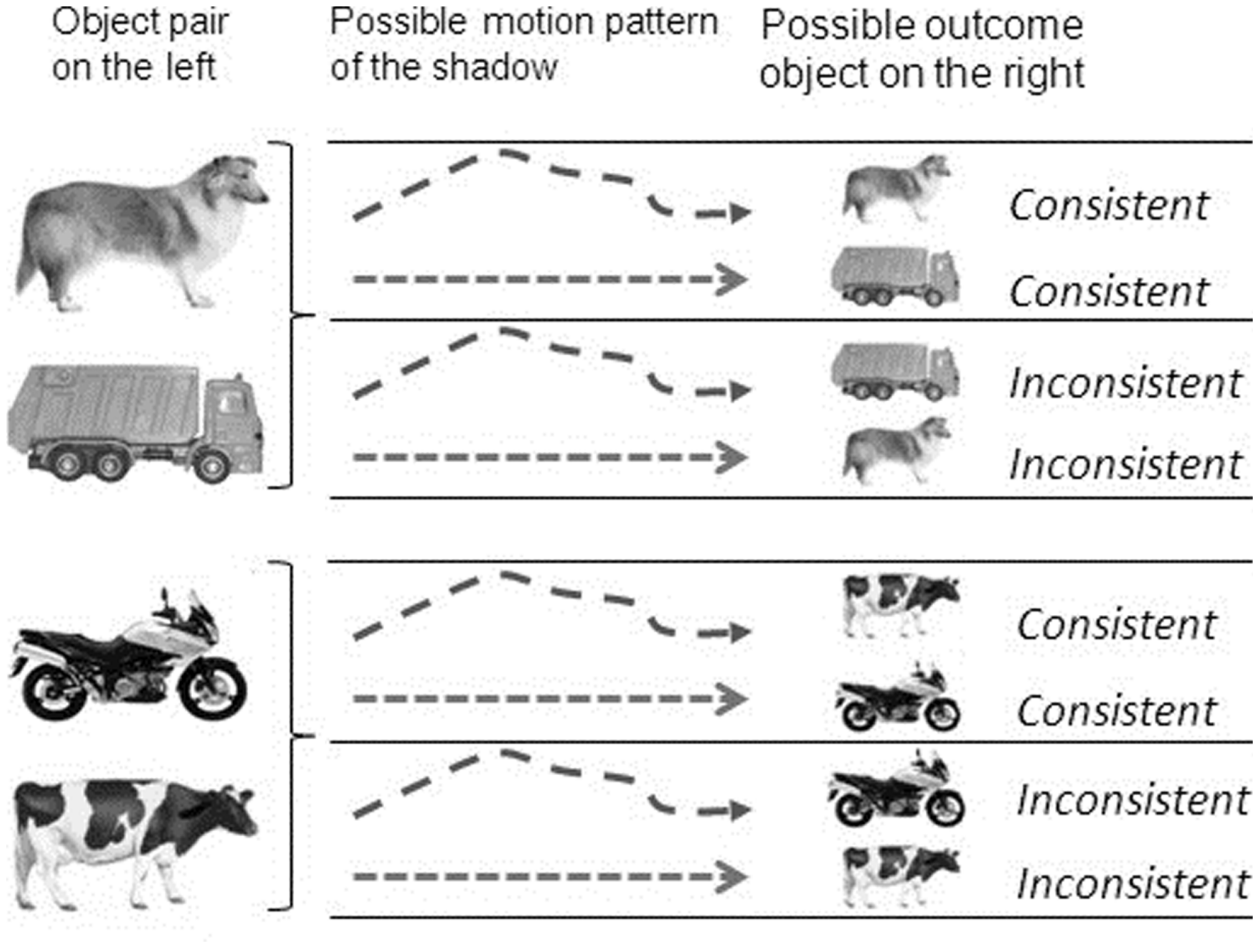
**Object-pairs, motion patterns and test-outcomes presented on each trial**.

Using a within-subjects design, each infant was administered four trials including both stimulus pairs (two consecutive trials including dog-truck, two consecutive trials including cow-motorcycle). The first two trials (Part A) as well as the last two trials (Part B) ended with the same outcome object. If the first two trials ended with the animate object of the respective object pair, then the last two trials ended with the inanimate object of the respective object pair and vice versa (e.g., trial 1 and 2 showed the dog at test, trial 3 and 4 showed the motorcycle). The type of motion pattern shown during the motion scene alternated between the four trials. Whether the first trial included an animate motion or an inanimate motion during the motion presentation phase, i.e., whether this motion pattern was consistent or inconsistent with the object shown at test, was counterbalanced across the entire sample. Each infant saw two consistent and two inconsistent test trials (see **Figure 1**).

### PROCEDURE

Infants were tested in a dimly lit room and sat in a high chair at a distance of 70 cm from the monitor. Presentations were shown on a 17″ TFT monitor and infants’ looking behavior was recorded by a Tobii T60 eye-tracker (TOBII Technology AB, Danderyd, Sweden).

**FIGURE 2:**
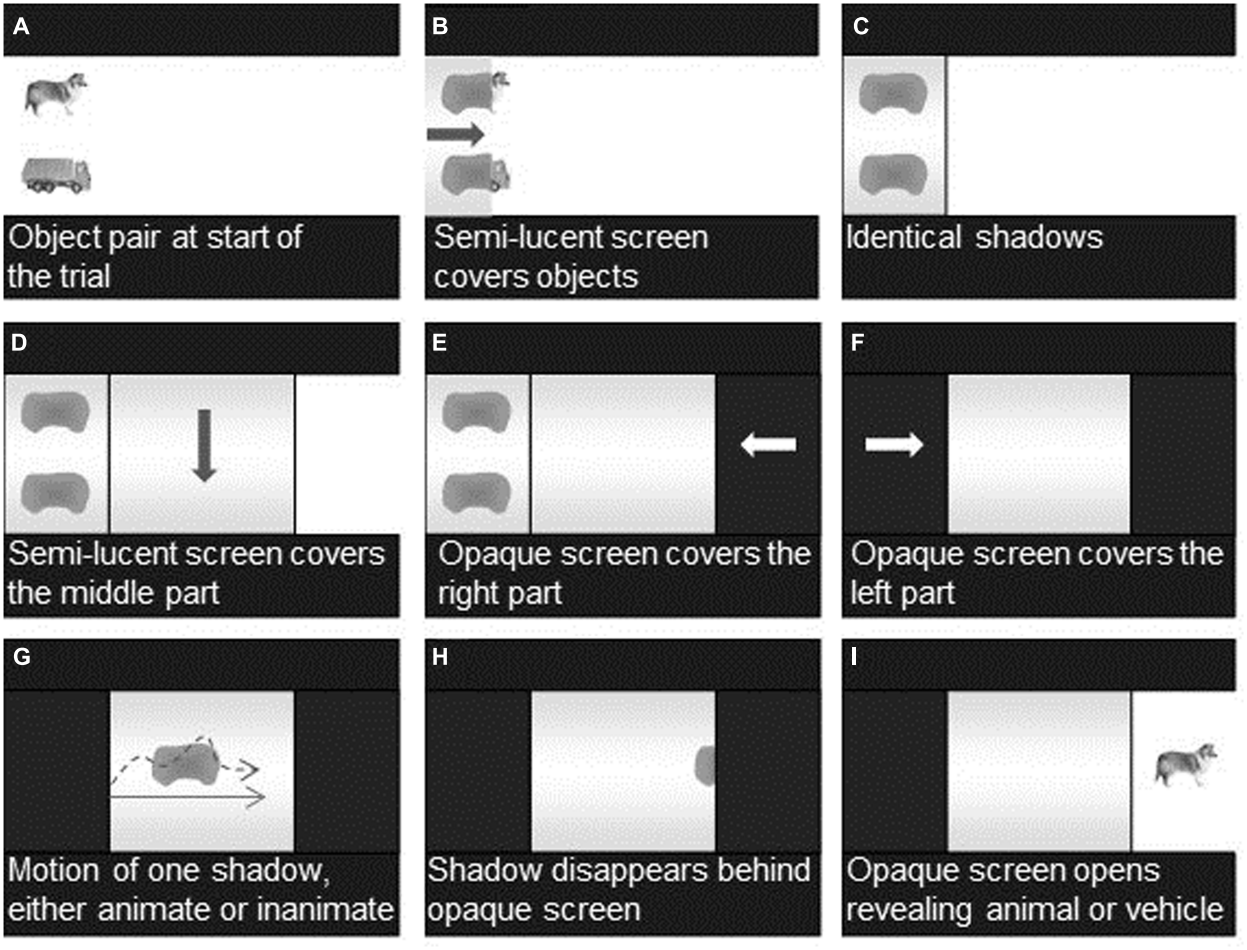
**Still frames from the computer animations used in the study.** Main sequences **(A–I)** are described in the Procedure section. Arrows are added to indicate direction of movement.

The stimuli (approximate size: 5.3 × 3.8 visual degrees) were presented in computer-animated scenes (32.5 × 12.9 visual degrees) each consisting of three phases. During phase 1, infants were shown both stimuli as well as three separate screens: two smaller screens covering 25% of the scene on the left and the right side of the display, respectively, and one screen covering the middle of the display (50%). At the beginning of each trial, infants first saw one pair of stimuli (e.g., dog-truck), placed above each other, both located on the left side of the display. During a 15-s time interval the two objects were covered and uncovered three times by a semi-lucent screen emerging from, and disappearing to the left side of the display. When covered, the two objects appeared as two identical shadows (see **Figures 2A–C**). After the third covering event with the left screen, another semi-lucent screen was lowered down to cover the middle section of the screen and remained in place. Next, an opaque screen emerged from the right side of the screen and covered the right side of the display, followed by an additional opaque screen entering from the left side overlaying the left semi-lucent screen and fully covering both shadows (see **Figures 2D–F**). At the end of phase 1, which lasted a total of 25 s, all three screens (two opaque and one semi-lucent) covered the display thus hiding the two stimuli as well as their shadows.

During phase 2 (motion phase) that immediately followed phase 1 and lasted for 10 s, infants saw one single shadow emerging from behind the middle of the left opaque screen, moving across the middle semi-lucent screen and disappearing behind the right opaque screen (see **Figures 2G,H**). The motion pattern was either animate or inanimate: The *animate motion pattern* included changes in speed constancy (4× motion changes) as well as direction changes (3× approximately 45° turns, starting with a 45° angle of climb), with the principal axis of the shadow always aligned with the direction of motion. These changes were evenly distributed across the 10-s motion phase so that infants who were attentive to the motion phase for 3 s or more saw at least one change in speed constancy and one direction change. Infants who did not meet this criterion were excluded from further analyses.

The *inanimate motion pattern* included a constant linear trajectory from the left to the right side of the scene. To ensure consistency across conditions, infants also needed to look for at least 3 s in the linear-motion presentations in order to be included in the final sample. Because the shadows for both the animal and the vehicle looked identical, static perceptual information could not be used to infer which of the two stimuli moved behind the semi-transparent screen. The only information available for making inferences about the object identity was its motion pattern.

Phase 3 (test phase) started once the shadow had disappeared behind the right opaque screen. The opaque screen then slid to the right (3 s) revealing either the animate or the inanimate entity presented during phase 1 (see **Figure 2I**). The corresponding stimulus was presented for 15 s. Depending on the motion pattern presented during phase 2 and the stimulus revealed during phase 3, the trials could either be consistent (i.e., an animal following the animate motion or a vehicle following the inanimate motion) or inconsistent (i.e., an animal following an inanimate motion, or an inanimate object following an animate motion).

The total duration of the presentation (all four trials) was about 3.5 min. For every given pair of stimuli (i.e., dog-truck, cow-motorcycle), each entity was shown once in the upper position and once in the lower position during the first phase. Each entity appeared equally often as test-exemplar and was equally often preceded by an animate or an inanimate shadow-motion during the motion phase.

The parent sat in a nearby chair and was asked not to interact with the infant. During the presentation, the experimenter was occluded by a curtain and monitored infants’ fixations via a video-camera. As soon as the infant’s attention was focused on the screen, the experimenter started the computer-controlled presentation. For the first 15-s period of each trial (phase 1) it was measured how long infants looked to the left 25% part of the screen, showing both objects one above the other. For each test phase (phase 3), an area of interest (AOI) was defined that covered the outcome-object (including an approximately 0.9 visual degree outline to account for sampling errors). Infant’s fixation duration to that AOI during the test phase was measured. Fixation was defined as stable gaze within one visual degree for at least 200 ms (e.g., Gredebäck et al., 2010).

### RESULTS AND DISCUSSION

The mean looking time during the first 15 s period showing the two objects before the semi-lucent screen covered them for the third time was 4.57 s (SE = 0.16). Infants had no *a priori* preferences for one of the two object pairs shown during phase 1 [dog-truck: *M* = 4.41 s, SE = 0.24; cow-motorcycle: *M* = 4.49, SE = 0.26; *t*(34) = -0.24, *p* = 0.81]. There was also no significant motion-type dependent difference in infants’ looking times during the motion phase (phase 2) with similar looking times at animate motion scenes (*M* = 6.22, SE = 0.21) compared to inanimate motion scenes (*M* = 6.08, SE = 0.22), *t*(34) = 1.35, *p* = 0.18.

For the test phase, an analysis of variance was conducted to test whether infants differentiated between test trials showing objects congruent or incongruent with the observed motion pattern of the shadow. Preliminary analyses revealed that neither the type of object (animate or inanimate objects at test), nor the order of presentation (consistent or inconsistent outcomes first) had any effect on infants’ looking responses during the test trials. A 2 × 2 repeated measures analysis of variance was conducted with *Congruency Type* (motion pattern consistent or inconsistent with test object) and *Motion Type* (animate or inanimate motion) as within-subjects factors. This analysis revealed a significant main effect for *Congruency Type F*(1,34) = 34.00, *p* < 0.001, η^2^ = 0.50, with longer looking times for inconsistent outcomes (*M*_inconsistent_ = 4.54, SE = 0.28) compared to consistent ones (*M*_consistent_ = 3.19, SE = 0.21). No other main effect or interaction was observed.

**FIGURE 3:**
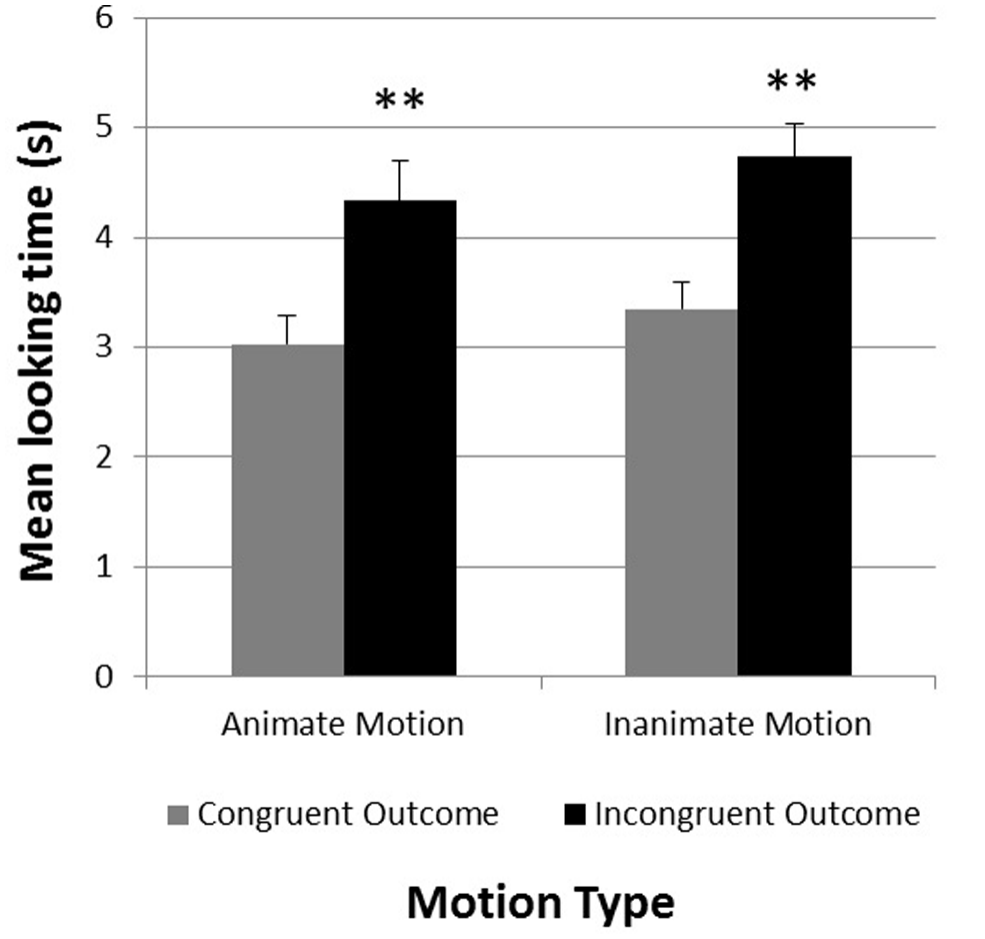
**Experiment 1.** Mean looking times in seconds during congruent and incongruent test trials (separated by motion type). **p < 0.01. Error bars represent +1 SE.

An examination of individual infants’ looking times confirmed these results. 30 of the 35 infants looked on average longer at the incongruent test trials compared to the congruent test trials [χ^2^(1) = 17.86, *p* < 0.001]. **Figure 3** shows the mean looking times for the congruent and incongruent test trials as a function of *Motion Type*.

As hypothesized, infants looked longer at test trials revealing inconsistency between appearance information and previous motion information regarding the target entity. This suggests that they were able to use markedly reduced dynamic information (changes in speed constancy and direction) to infer the animacy status of the previously shown morphologically ambiguous shadow. However, in the present study, infants always saw both kinds of objects as static pictures at the beginning of the presentation, followed by the dynamic displays, and ending up with one of the static picture. Hence we cannot rule out that infants benefited by *a priori* static information to process the motion information.

The aim of Experiment 2 was to clarify the relative impact of given static information by replicating Experiment 1 without presenting the two objects before the moving shadow.

## EXPERIMENT 2

### PARTICIPANTS

A total of 24 7-month-old infants (*M* = 7 months, 14 days; range = 7 months, 1 day to 7 months, 30 days), 10 girls and 14 boys participated. Eight additional infants had to be excluded due to fussiness (*n* = 3), or failing to look during phase 3 during all four test trials (*n* = 5). All infants came from a White, middle-class socioeconomic background.

### MATERIAL AND PROCEDURE

Material, Design, and Procedure were the same as in Experiment 1, with the only difference that there was no presentation of the two static pictures prior to the shadow’s motion. Consequently, the presentation started by showing an opaque screen covering the left side of the display. A semi-lucent screen was lowered down to cover the middle section of the screen and remained in place, followed by an opaque screen emerging from the right side and covering the right quarter of the display. Next, the motion phase started: One single shadow emerged from behind the middle of the left opaque screen, moved across the middle semi-lucent screen and disappeared behind the right opaque screen. The sequence of motion patterns (animate and inanimate patterns) as well as the duration of the motion phase was identical to Experiment 1. The following test phase was also equivalent to the first experiment by removing the right opaque screen revealing either an animate or an inanimate entity for 15 s.

As in Experiment 1, each infant saw two consistent test trials (an animal following the animate motion pattern or a vehicle following an inanimate motion pattern) and two inconsistent test trials (an animal following the inanimate motion pattern or a vehicle following an animate motion pattern). The total duration of the presentation (all four trials) was about two minutes.

### RESULTS AND DISCUSSION

There was no significant motion-type dependent difference in infants’ looking times during the motion phase (phase 2) with similar looking times at animate motion scenes (*M* = 6.16, SE = 0.26) compared to inanimate motion scenes (*M* = 5.96, SE = 0.24), *t*(23) = 1.18, *p* = 0.25.

For the test phase, an analysis of variance was conducted to test whether infants differentiated between test trials showing objects congruent or incongruent with the observed motion pattern of the shadow. Preliminary analyses revealed that neither type of object (animate or inanimate objects at test), nor order of presentation (consistent or inconsistent outcomes first) had any effect on infants’ looking responses during the test trials.

**FIGURE 4:**
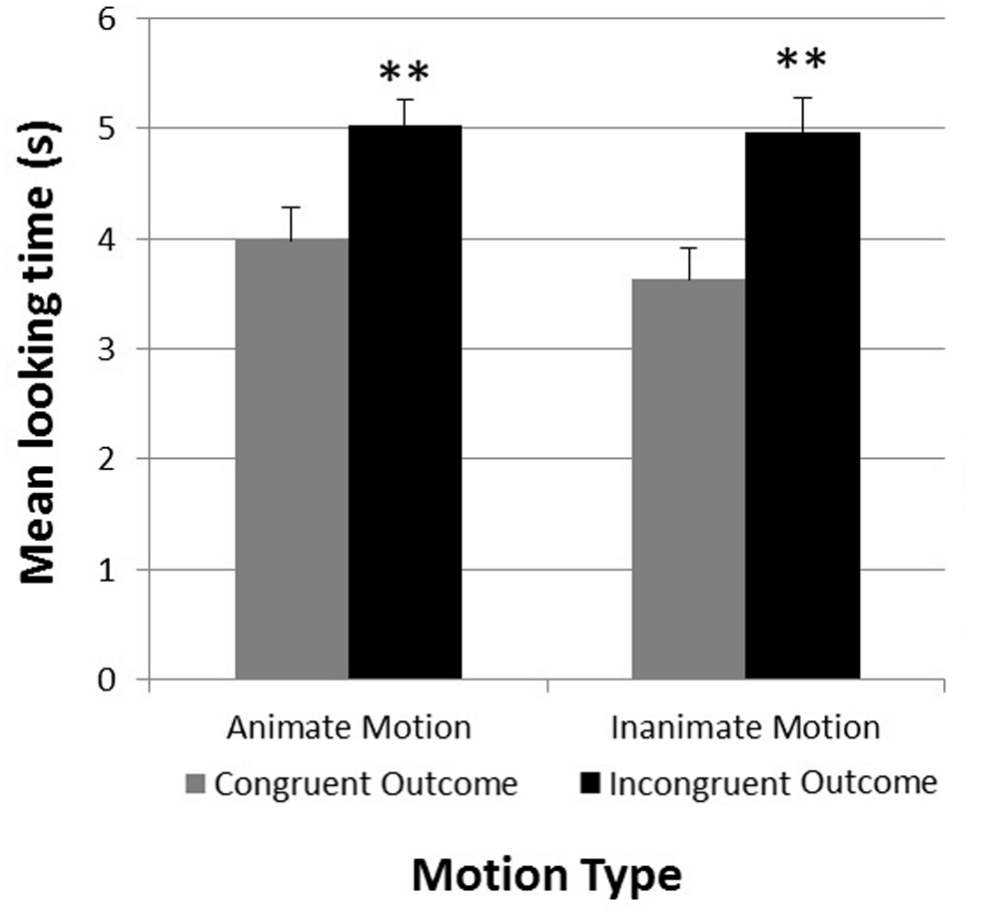
**Experiment 2.** Mean looking times in seconds during congruent and incongruent test trials (separated by motion type). **p < 0.01. Error bars represent +1 SE.

A 2 × 2 repeated measures analysis of variance was conducted with *Congruency Type* (test object consistent or inconsistent with motion pattern) and *Motion Type* (animate or inanimate motion) as within-subjects factors. This analysis revealed a significant main effect for *Congruency Type F*(1,23) = 29.98, *p* < 0.001, η^2^ = 0.50, with longer looking times for inconsistent outcomes (*M*_inconsistent_ = 4.99, SE = 0.23) compared to consistent ones (*M*_consistent_ = 3.81, SE = 0.26). No other main effect or interaction was observed. An examination of individual infants’ looking times confirmed these results. 0.21 of the 24 infants looked on average longer at the incongruent test trials compared to the congruent test trials [χ^2^(1) = 13.50, *p* < 0.001]. **Figure 4** shows the mean looking times for the congruent and incongruent test trials as a function of *Motion Type*.

In conclusion, infants’ looking pattern in Experiment 2, presenting no static pictures of an animal and a vehicle prior to the shadow’s motion, does not differ from the results obtained in Experiment 1. On the basis of these data, it seems unlikely that the static pictures shown at the beginning of each trial in Experiment 1 played a key role in infants’ detection of category-motion incongruencies at test. This suggests that longer looking times during the inconsistent test trials in Experiment 1 reflected the recognition of category-specific motion cues without any form of priming.

## GENERAL DISCUSSION

The main goal of the current experiments was to determine if infants have already formed specific expectations about the type of motion cues that have been found to generate animacy judgments in older children and adults. The present results suggest that 7-month-olds’ representations of animals and vehicles include category-specific associations between static and dynamic cues that are considered minimal conditions for animacy perception (e.g., Scholl and Tremoulet, 2000; Tremoulet and Feldman, 2000; Schultz and Bülthoff, 2013).

These findings suggest that within their first year of life, infants have already formed some stable associations between static and dynamic attributes characterizing global categories. However, while Arterberry and Bornstein (2002) provided relevant evidence with 9-month-olds in a task where dynamic information was provided via point-light displays, displaying manner of motion as well as implicit shape information (e.g., Cutting, 1978; Giese and Poggio, 2003; Schlottmann and Ray, 2010) of the objects, the present study manipulated the motion cues of ambiguous shapes so that no morphological cues would allow infants to identify either an animal or an inanimate object. While Pauen and Träuble (2009) also tested 7-month-olds, their task provided infants with static and dynamic information simultaneously. In contrast, we attempted to dissociate dynamic and static information.

Referring to Arterberry and Bornstein (2002), it has been shown that 9-month-olds, but not 6-month-olds, succeed in a categorization task presenting dynamic point-light displays for habituation and static images as test stimuli. According to the authors, this reflects that with age, infants become less tied to specific stimulus information with age, and show the ability to transfer category knowledge across cues. It should be noted, though, that 9-months-olds were able to transfer animacy-specific expectations from dynamic to static displays but not vice versa.

One might speculate that it was easier for infants tested with the Arterberry and Bornstein (2002) paradigm to extract the implicitly given shape information from the dynamic point-light displays presented over several trials during familiarization and to make the transfer to a static picture at test. On the contrary, familiarized with a series of static pictures, the abstraction of shape from a point-light display at test seemed much harder.

Such a dissociation between dynamic and static feature processing is in accordance with the fact that both types of information are processed along different neural pathways (Leslie et al., 1998; Mareschal et al., 1999; Mareschal and Johnson, 2003). It is well established that, from birth, motion helps infants to first direct their eye gaze toward the object by triggering subcortical pathways (colliculi superiores). Once the object is in focus, in depth encoding of specific parts or features can take place. Research on early categorisation also suggests that information about motion and/or function relevant object parts facilitate object classification (e.g., Rakison and Cohen, 1999; Träuble and Pauen, 2007, 2011)

In comparison to Arterberry and Bornstein (2002), 7-month-olds participating in the present study succeeded in ascribing animacy-specific motion cues performed by a fully ambiguous shape to subsequently presented static pictures of the corresponding objects. While in the Experiment 1 infants always saw an exemplar of both kinds of objects as static pictures at the beginning of each trial, Experiment 2 yielded the same results without providing any *a priori* static information before the start of the shadow movement. This suggests that *a priori* given static information is not necessary for establishing animacy-specific relations between dynamic and static features.

Previous research on infants’ knowledge of properties of animates and inanimates based on dynamic information typically focused on specific motion patterns like rigid versus non-rigid (caterpillar) motion. The present study extends this line of research by showing that in addition to motion pattern, motion path is also an important and early accessible cue to animacy status. Until today, the nature of early animacy-ascriptions to given events or entities is not fully clarified. While some authors interpret corresponding data as a sign for conceptual or higher level processes (e.g., Arterberry and Bornstein, 2002; Pauen and Träuble, 2009), others assume that ascriptions of animacy or causality to given events might be to a large extent perceptual in nature (e.g., Scholl and Tremoulet, 2000). The mere fact that the ascription of animacy or causality can be mediated by highly reduced motion cues in simple displays (e.g., Leslie, 1986; Blythe et al., 1999; Tremoulet and Feldman, 2000; Schultz and Bülthoff, 2013) led to the assumption that such phenomena reflect first and foremost perceptual, perhaps modular processes that are clearly distinguishable from higher-level cognitive analyses. Scholl and Tremoulet (2000) suggest that such phenomena might lie at an intersection of perceptual and cognitive processing (see also Leslie, 1986 for a proposal on a modular based mechanism for interpretations of events as being causal).

The present set of data cannot resolve this issue. It might be that the motion cues provided in the present task are processed within an automatic encapsulated perceptual mechanism, resulting in an impression of animacy that either does or does not match with conceptual representations activated by the outcome picture presented at test. Furthermore, the matching process at test might also be mainly perceptual in nature, reflecting perceptually based associations between animacy-specific dynamic and static features. Such an assumption conflicts with more conceptual accounts that assume that higher-level processes determine the animacy status of an entity, including various assumptions on the nature and content of higher-level mechanisms that might be involved in conceptual information processing (e.g., Keil, 1991; Gelman et al., 1995; Keil et al., 1998; Mandler, 2004). Regardless of the automaticity of this visual phenomenon, the fact that individual variability in sensitivity to animacy cues predicts social cognition (e.g., empathy) in adults suggests that it might represent a building block in the development of more conceptual representations of social stimuli (Miller and Saygin, 2013).

In any case, we conclude that transfer performances as shown by 7-month-old infants in the present task and also by slightly older infants in the study of Arterberry and Bornstein (2002) include processes going beyond the perceptual information currently present in a given event. Seven-month-olds in the current study treated animacy-specific static and dynamic features as belonging together even when both types of features were presented in isolation. Rakison and Poulin-Dubois (2001, 2002) as well as Pauen and Träuble (2008) adopt a more moderate definition of conceptual processing, including the ability to form lasting relations between animacy-specific dynamic and static information in combination with the ability to activate according relations when only one type of information is given. In this sense, infants’ looking patterns at test might reflect the activation of conceptual representations of animate and inanimate entities. To determine the role of conceptual processes that might be involved in these early inferences, further research is needed.

In sum, the current results obtained with an innovative paradigm are in line with the assumption that 7-month-old infants are able use specific dynamic cues, known to trigger animacy perception in adults, to infer the identity of morphologically ambiguous entities. Future research with this paradigm can clarify whether infants draw similar inferences about animacy from a wide range of motion cues.

## Conflict of Interest Statement

The authors declare that the research was conducted in the absence of any commercial or financial relationships that could be construed as a potential conflict of interest.
